# A program to reduce self-stigma in patients with endogenous psychiatric disorders

**DOI:** 10.1192/j.eurpsy.2025.765

**Published:** 2025-08-26

**Authors:** V. Mitikhin, P. Orekhova, L. Alieva

**Affiliations:** 1 Mental Health Research Centre, Moscow, Russian Federation

## Abstract

**Introduction:**

Stigmatization and self-stigmatization remains an actual problem for patients with endogenous mental illnesses, as it is an obstacle to seeking psychiatric help. Taking this into account, it is necessary to develop effective psychosocial interventions aimed at reducing self-stigmatization and improving patients’ integration into society.

**Objectives:**

To identify the features of self-stigmatization in patients with schizophrenic spectrum disorders and, taking into account these features, to develop a program to reduce it.

**Methods:**

30 patients with schizophrenic spectrum disorders (F20, F23, F25 according to ICD-10) were included in the study. The average duration of the disease was 13.5±3.2 years. Among them, 14 were males, 16 were females, and the median age of the patients was 42.21±10.36 years. To assess the severity of self-stigmatization and to determine its components, the “Questionnaire for assessing the phenomenon of self-stigmatization in psychiatric patients” (Yastrebov V.S., Mikhailova I.I., Yenikolopov S.N. et al., 2005) was used.

**Results:**

A rather high general level of self-stigmatization (1.20±0.57 points) was revealed in the studied patients, exceeding the average indices according to the mentioned questionnaire. The most pronounced were the indicators on the following scales: “Overestimation of inner activity” (1,61±0,67 points); “Overestimation of self-actualization” (1,48±0,78 points); “Willingness to distance from mentally ill patients in the social sphere” (1,44±0,72 points); “Violation of self-identity” (1,17±0,59 points). Taking into account the identified disorders, a program including psychoeducation, as well as art-therapeutic training based on the approach of Z. Russinova et al. “Anti-stigma photovoice Intervention” (2014) was developed and adapted for the Russian population. The psychoeducation included three sessions where the manifestations of mental disorders, their treatment, forms of psychiatric care, issues of stigmatization and its overcoming were discussed. The training included six sessions discussing the following topics: “My daily life”, “Health and illness”, “Me and others”, “Accepting help and giving help”, “My achievements and my possibilities”, and “The next chapter of my life”. Participants provided pictures according to the session topic and discussed personal experiences, their emotions and feelings. The sessions were held in a closed group, the number of participants from 8 to 12 people, and the duration of the session was 90 minutes.

**Conclusions:**

The developed program contributed to the identification of resources that help in overcoming the disease and reducing self-stigma. The program can be used for patients in the initial stages of the disease and with a long-term course of the disease.

**Disclosure of Interest:**

None Declared

